# Prognostic Implications of PET-Derived Tumor Volume and Uptake in Patients with Neuroendocrine Tumors

**DOI:** 10.3390/cancers15143581

**Published:** 2023-07-12

**Authors:** Manuel Weber, Tugce Telli, David Kersting, Robert Seifert

**Affiliations:** Department of Nuclear Medicine, University of Duisburg-Essen and German Cancer Consortium (DKTK)-University Hospital Essen, 45147 Essen, Germany

**Keywords:** somatostatin receptor, NET, PET, tumor volume, SUV

## Abstract

**Simple Summary:**

The monitoring of disease in patients with neuroendocrine tumors is challenging and requires specialized imaging techniques. To address these quests, somatostatin receptor-targeting molecular imaging modalities that can accurately visualize disease manifestations of neuroendocrine tumors have been proposed. However, the prognostic value of the total tumor volume, expression level, and related metrics has yet to be thouroughly understood.

**Abstract:**

Historically, molecular imaging of somatostatin receptor (SSTR) expression in patients with neuroendocrine tumors (NET) was performed using SSTR scintigraphy (SRS). Sustained advances in medical imaging have led to its gradual replacement with SSTR positron-emission tomography (SSTR-PET). The higher sensitivity in comparison to SRS on the one hand and conventional cross-sectional imaging, on the other hand, enables more accurate staging and allows for image quantification. In addition, in recent years, a growing body of evidence has assessed the prognostic implications of SSTR-PET-derived prognostic biomarkers for NET patients, with the aim of risk stratification, outcome prognostication, and prediction of response to peptide receptor radionuclide therapy. In this narrative review, we give an overview of studies examining the prognostic value of advanced SSTR-PET-derived (semi-)quantitative metrics like tumor volume, uptake, and composite metrics. Complementing this analysis, a discussion of the current trends, clinical implications, and future directions is provided.

## 1. Introduction

Neuroendocrine tumors (NETs) encompass a variety of tumors that most commonly originate from the gastrointestinal system, the pancreas, and the lungs [1,2,3,4,5,6,7,8,9,10,11]. Their incidence is relatively low, but a steep increase has been noted over the past decades (1973: 1.09/100,000: 2004, 5.25/100,000) [2,12]. Up to 85% of patients are initially metastatic, drastically reducing the likelihood of achieving complete remission [13,14,15]. Somatostatin receptor (SSTR)-directed molecular imaging, targeting especially SSTR subtype 2, which is highly expressed in NETs, has shown high sensitivity [16,17,18,19,20,21,22,23,24,25,26,27,28,29,30,31,32,33]. The most accurate imaging modality in this regard appears to be SSTR-directed positron-emission tomography (SSTR-PET), which has shown high sensitivity, often outperforming both conventional cross-sectional imaging like MRI and CT and somatostatin receptor-directed scintigraphy (SRS) [34,35,36,37,38,39,40,41,42,43,44,45,46,47,48,49,50,51,52,53,54,55,56]. This promising diagnostic performance has been shown to alter subsequent treatment decisions and the management of patients [12,24,30,57,58,59,60,61,62,63,64,65,66,67]. However, it must be noted that highly proliferative neuroendocrine tumors may present negatively on SSTR-directed imaging due to the loss of SSTR expression.

In addition, SSTR-targeting radiopharmaceuticals can not only be used for imaging purposes but also for therapy. In a personalized theragnostic treatment approach, patients with strong expression of the target molecule, as assessed by molecular imaging, are selected for therapy. Following this concept, SSTR-directed imaging is routinely performed before SSTR-targeted therapies, such as peptide radionuclide receptor therapy (PRRT) [68,69,70,71]. The traditionally performed SRS has, however, fallen out of favor due to its inferior diagnostic performance compared to SSTR-PET [39,72]. In this context, it is worth pointing out that the higher sensitivity of SSTR-PET does not only translate to a higher detection rate but also has relevant implications for the quantification of SSTR expression. In particular, small lesions can visually show increased signals when compared to the reference tissue on SSTR-PET vs. SRS, which has been shown in a head-to-head comparison of NET patients undergoing both imaging modalities [73]. Because of this effect, commonly employed criteria to assess subject eligibility for PRRT cannot be directly translated from SRS to SSTR-PET, and the need for new quantitative parameters to assess treatment suitability, predict response, and prognosticate the outcome is raised. Therefore, a comprehensive framework to assess therapy SSTR-directed radioligand eligibility using SSTR-PET is required.

The superior spatial resolution of SSTR-PET, together with advances in functionalities and increased availability of segmentation tools, enables PET-derived quantification of the whole-body tumor volume. Thus, qualitative and quantitative prognostic markers can be integrated into the management of patients, such as tumor burden (quantified by somatostatin receptor-expressing tumor volume, SRE-TV), SSTR expression levels, as well as other more complex markers, such as heterogeneity or sphericity [74,75]. The use of these PET parameters in conjunction with clinical and laboratory data may allow for the creation of nomograms and, thereby, aid treatment decisions by identifying patients who are most likely to not respond to PRRT. Similar approaches have been published in the context of PSMA-radioligand therapy for prostate cancer [76]. In patients most likely to not respond to PRRT, the use of alternative treatment strategies may be preferable. 

The published evidence on the prognostic value of SSTR-PET-derived volumetric parameters is steadily growing and current developments seem promising. Most of these studies were able to show a prognostic impact of SRE-TV with regard to relevant patient outcomes, such as treatment response, progression-free survival (PFS), and overall survival (OS), both in patients with varying post-SSTR-PET treatment, but also in patients undergoing specific, often SSTR-directed treatment such as somatostatin analogs (SSA). The aim of this narrative review is to provide an overview of the published literature on SSTR-PET-derived volumetric parameters in NETs, with a focus on the methodology employed and the relevance of patient outcome prognostication. 

## 2. Methodology of Literature Research

This narrative review aims to assess the prognostic value of SRE-TV and related quantitative metrics in NET patients across published original articles. Studies were identified using a structured PubMed search using the terms “Neuroendocrine PET volume” (search date: 13 December 2022). Articles were excluded based on the following basis: 53 no PET-based volumetric assessment, 32 no neuroendocrine tumors in the collective, 13 reviews, 5 case reports, and 7 preclinical studies. A total of 143 trials were found in the initial database search, and 32 articles were eligible. To provide a comprehensive overview and focus on SSTR-PET-derived tumor volume, we also discuss studies on SSTR expression and FDG-PET-derived features.

## 3. Role of SSTR-PET-Derived Biomarkers for the Management of NET Patients 

### 3.1. Tumor Volume Measurement Techniques

Different methodologies for whole-body tumor volume measurements using both SSTR-PET (SRE-TV) and FDG-PET (metabolic tumor volume, MTV) have been described in the literature. Most of these methodologies fall under one of the following categories:

(i) One fixed global threshold for all tumor lesions across patients.
(ii) Patient-specific thresholds based on uptake in reference organs, such as blood pool, liver, or spleen.
(iii) Lesion-specific local thresholds (isocontour), based on the SUVmax of the respective lesion.
(iv) Manual measurements without thresholding. These approaches may limit interobserver agreement based on the high degree of freedom that each observer has for lesion delineation.
(v) Combinations of i/ii and iii, in which a global or patient-specific threshold is used to preselect lesions, which are thereafter segmented with a local isocontour.

A fixed global threshold for tumor volume measurement has only been explicitly employed in a few FDG-PET-based studies, where threshold SUVs of 2.5 and 4 were used for MTV assessment [77,78].

### 3.2. Tumor Volume in the Context of SSTR-Targeted Imaging

For SRE-TV measurements, the most commonly used reference organs are the liver and the spleen. Despite this commonality in approach, the methodologies show slight deviations.

Ortega et al. have used the SUVmax of a three-dimensional volume of interest, both for the liver and the spleen as a threshold for the definition of SRE-TV [79]. Influenced by the PERCIST criteria, a threshold of 1.5 × SUVmean + 2 × SD (standard deviation) of healthy liver tissue in a 3 cm diameter VOI has been employed as well [80,81,82]. When using the spleen uptake as a reference, the following formula can be used instead: 0.67 × SUVmean + 2 × SD [82].

The use of local thresholds has commonly been employed by various investigators, for both MTV and SRE-TV.

The reported thresholds for MTV determination are 40% [83], 45% [84], and 50% [85,86].

The earlier adoptions of this methodology for SRE-TV have led to the use of often (seemingly) arbitrary thresholds, often derived from FDG-PET studies on various tumor entities.

Ohnona et al. used a 41% threshold of the lesional SUVmax in a study of 50 patients with G1/2 pancreatic NET (pNET) [87]. The same approach was later used by Durmo et al. [88], although 40% [89] and 50% [86,90,91] thresholds were used as well. 

Liberini et al. compared different segmentation thresholds (20% vs. 30% vs. 40%) with regard to their robustness for the assessment of radiomics features and concluded that a local threshold of 40% produced the most reliable results [92]. 

A head-to-head comparison of manual and threshold-based SRE-TV (50%, 42%, and subtracted background activity) measurements using morphological tumor extent as the gold standard showed that a threshold of 42% had the strongest correlation with morphological tumor volume and least bias [93]. A different study that compared local thresholds with thresholds based on reference organ uptake showed a higher correlation between local threshold-derived SRE-TV and patient outcome, with a 30% threshold being the most accurate predictor [94]. 

In addition, it has been shown that SRE-TV measurements are affected by image reconstruction algorithms, such as the point spread function [95] and gated image acquisition, particularly in body areas that are most affected by respiratory motion [96]. 

### 3.3. Association of SSTR-PET-Derived Tumor Volume with Clinical Outcome

Tumor burden, parametrized by SRE-TV, has shown a weak association with specific hormone-associated symptoms, such as diarrhea, flushing, and dyspnea [90]. At the biochemical level, this may be attributable to markers associated with hormonal activity, such as chromogranin A, hydroxy indole acetic acid, and—in the specific case of von Hippel Lindau syndrome—vasointestinal peptide and polypancreatic peptide, for which a positive correlation SRE-TV has been shown [86,97]. 

Investigators from the same group also found that SRE-TV was inversely correlated with PFS in an analysis of patients with locally advanced or metastatic disease. In addition, a subgroup analysis of patients with pNET showed a negative prognostic impact of high (35.8 mL) SRE-TV (log-rank test, HR 16.4, *p* = 0.01) with regard to disease-specific survival, which was confirmed in a subgroup analysis of all patients without metastatic disease using both uni- and multivariate analyses. No statistically significant results were observed for small intestinal NETs (*p* = 0.1) [98]. Similarly, Ohnona et al. and Toriihara et al. observed that SRE-TV was an independent predictor of PFS in a cohort of 50 (*p* < 0.001) and 92 (*p* = 0.036) NET patients, respectively, who would later undergo different treatments, such as SSA therapy or surgery [87,91]. In a cohort of NET patients, which was more homogeneous in terms of treatment, Chen et al. showed that patients with non-functional NET and Ki67-index < 10% treated with SSA, SRE-TV, and liver-specific SRE-TV (each *p* < 0.02) were highly predictive of short PFS, whereas the SSA dose normalized to total and liver-specific SRE-TV was positively associated with PFS [94]. The latter was corroborated by Kim et al. in a cohort of 31 patients with well-differentiated gastroenteropancreatic NETs (GEP-NETs) undergoing SSA, where SRE-TV > 58.9 mL was associated with shorter PFS in the uni- but not in the multivariate analysis, whereas a higher tumor–liver ratio was significantly associated with longer PFS, even in the multivariate analysis (Hazard ratio 3.182, *p* = 0.02) [81]. In 20 patients with resectable pNETs, both SUV and total lesion SSTR expression, i.e., the product of SRE-TV and SUVmean, were highly correlated with locally advanced disease (*p* = 0.0002). Similarly, SUVmean of the entire SRE-TV (liver uptake was used as the threshold) showed an association with PFS (*p* = 0.0053) [79]. 

### 3.4. Association of PET-Derived SSTR Expression with Clinical Outcomes

SSTR expression is a critical component of NET pathogenesis and is associated with tumor proliferation and the degree of differentiation. As such, the quantification of SSTR expression through imaging has become an essential tool for the diagnosis and management of NETs, particularly for patients undergoing PRRT or SSA therapy.

Several studies have investigated the relationship between SSTR expression and tumor proliferation using SSTR-PET. A statistically significant negative correlation between the Ki-67 index and SUVmax on 68Ga-DOTATATE-PET was found after investigating 238 NET patients, out of which a subgroup of 54 patients had available histology (r = −0.3, *p* = 0.018) [99]. Simsek et al. reported that the median SUVmax was significantly higher in grade 1 in comparison to grade 2 NETs (23 vs. 17), grade 2a in comparison to grade 2b NETs (19.7 vs.9), and grade 1 in comparison to grade 2b NETs (23 vs.9) (*p* = <0.001, <0.001, and <0.05, respectively) [63].

A low SUVmax on SSTR-PET was found to be associated with poor OS in patients with different NET grades and with poor PFS in patients especially in patients with grade 1-2 NETs, independent of the type of 68Ga-DOTA-conjugated somatostatin analog (68Ga-DOTATATE, -NOC, or -TOC) or primary tumor site [100]. Moreover, because of SSTR-PET findings, intended management changes were reported in up to 60% of patients [12,101]. In patients whose Ki-67 index was lower than 12%, 68Ga-DOTATATE made a particularly larger contribution to clinical management than 18F-FDG PET. 

### 3.5. Association of SSTR-PET-Derived Parameters and Outcomes after SSA or PRRT

In contrast to FDG and tumor aggressiveness, SUVmax on SSTR-PET is associated with a lower grade, lower proliferation rate, and therefore, tumor phenotypes that are better controlled by SSA therapy. Different semi-quantitative parameters and their prognostic importance in patients treated with PRRT or SSA have also been analyzed. 

Campana et al. stated that the SUVmax of the highest uptake lesion on SSTR-PET was higher in patients with stable disease or partial response in comparison to patients with progressive disease at follow-up after PRRT or SSA. The best cut-off for the differentiation between those groups ranged from 17.6 to 19.3 (AUC: 0.75, *p* = 0.004), and values higher than 19.3 facilitated the selection of patients with a slow disease progression. Moreover, an SUVmax of 19.3 or more could be considered an index of a better prognosis in patients receiving PRRT or SSA on univariate and multivariate analyses [102]. Kratochwil et al. proposed an SUVmax cut-off of >16.4 in liver metastases on SSTR-PET to select patients for PRRT [69]. 

However, SUVmax depends on single voxel values and may be influenced by artifacts or the partial volume effect. Therefore, different measurement techniques (like SUV-mean) and ratios have been introduced to increase accuracy and implement scanner-independent SSTR-PET-based biomarkers. Koch et al. found that SUVmean and SUVmax of NET tumor lesions in SSTR-PET were associated with response to therapy with octreotide acetate. Kratochwil et al. recommended a tumor-to-liver ratio (TLR) higher than 2.2 as a cut-off value for the selection of patients to undergo PRRT [103]. A cut-off for the SUVmax of 29.4 and the SUVmean of 20.3 on SSTR-PET could distinguish between patients with a long PFS (69.0 weeks; 95% CI 9.8–128.2) and a short PFS (26.0 weeks; 95% CI 8.7–43.3) after octreotide acetate therapy [103]. In another study, low baseline SUVmean (≤11.2), elevated baseline inflammation-based index (IBI) derived from C-reactive protein and albumin, and TV (>672 mL) were independently associated with worse survival in NET patients treated with PRRT [104]. Another study of 91 patients who underwent SSTR-PET prior to PRRT showed a statistically significant association between the mean SUVmax of target lesions, TLR, SUVmax target lesion/spleen, and SUV-mean and progression or death (*p* =  0.002, 0.03, 0.04, and 0.005, respectively) [79]. For this analysis, up to 5 target lesions with a maximum of 2 per organ were selected based on reproducibility, delineation, and a minimum size of 1 cm. Furthermore, a tumor-to-liver ratio lower than 8.1 on SSTR-PET was found to be a statistically significant parameter associated with shorter PFS under lanreotide therapy in the multivariate analysis (hazard ratio 3.182 [95% CI 1.189–8.514], *p* = 0.021). The mean PFS intervals according to the tumor-to-liver ratio (<8.1 vs. ≥8.1) were 10.8 months (95% CI 5.8–15.8) and 23.0 months (95% CI 16.8–29.3), respectively [81].

Lastly, intra-tumoral heterogeneity and its importance in patients who received PRRT were found to be associated with outcomes. Graf et al. recently reported that the visual assessment of intra-tumoral SSTR heterogeneity had both predictive value and prognostic value in progressive grade 1 or grade 2 NET patients undergoing PRRT, exceeding the prognostic value of the Ki-67 index [105]. Visual analysis was performed for each lesion by looking for regions inside the lesion that would have an uptake intensity greater than the maximum uptake of the lesion. Patients with heterogeneous SSTR expression have a 3.7 times higher risk of death than patients with homogeneous SSTR expression.

### 3.6. Association of FDG-PET-Derived Features with Clinical Outcomes

Grade 1 (G1) NET tumors typically demonstrate neuroendocrine cell characteristics, such as high levels of SSTR expression and secretion of variable hormones comparable to normal neuroendocrine cells. On the other hand, the higher the grade, the poorer the differentiation levels and proliferative rates, which are associated with higher glycolytic metabolism of tumor cells [106]. In this continuum of decreasing SSTR expression and increasing FDG uptake, various intermediate stages can be observed: FDG-positive lesions were reported in 40% of G1, 50% of G2, and 93% of G3 NET patients [107]. Panagiotidis et al. reported that SSTR and FDG-PET findings were discordant in 62.5% of the NET patients. The results of FDG-PET did not change the therapeutic plan for G1 patients, but had a moderate impact on G2 patients (and a high impact on poorly differentiated NETs). Moreover, a significant positive correlation was noted between Ki-67 and SUVmax on FDG-PET (*p* = 0.002) [101]. In another study of 27 NET patients, SUVmax on FDG-PET correlated with greater tumor size, higher expression of Ki-67, and lower expression of the Von Hippel Lindau gene, whose inactivation leads to the accumulation of the hypoxia-inducible factor protein and “pseudohypoxic” state with enhanced transcription of specific target genes, such as CA9 and GLUT1 (*p* = 0.03, 0.004 and 0.008, respectively) [108]. 

Interestingly, FDG positivity is not rare in metastatic well-differentiated G1/G2 NETs and can be a stronger predictor of progression and prognosis than the existing WHO groups. In a study of 495 NET patients who were referred to PRRT, median OS, and PFS were significantly higher in the FDG-negative than in the FDG-positive group (83.1 months vs. 53.2 months, *p* < 0.001) and PFS (24.1 months vs. 18.5 months, *p* < 0.002). In this study, FDG-positive lymph node and liver tumor burden were also independent predictors of OS (*p* = 0.035 and *p* = 0.034), whereas FDG-avid bone tumor burden (metastases) was an independent predictor of PFS (*p* = 0.001). Likewise, median OS and PFS were significantly higher in the FDG-negative group than in the FDG-positive group (median OS: 97.7 months vs. 51.0 months, *p* = 0.01; median PFS: 33.8 months vs. 19.9 months, *p* = 0.05) [109].

The prognostic importance of different semi-quantitative parameters on FDG-PET has been investigated in different studies. In a study of 89 NET patients, SUVmax > 3 on FDG-PET was found to be related to lower PFS (HR: 8.4; *p* < 0.001), while SUVmax > 9 was associated with lower OS (HR: 8.8 95% CI, 2.7–28.7, *p* = <0.001) [107]. In another study of 89 patients with metastatic inoperable gastroenteropancreatic NET, the ratio of SUVmax of the tumor with the highest SUV to the SUVmax of the normal liver parenchyma (TLR) was calculated using FDG-PET. TLR > 2.3 was an independent predictor of poor outcome, with an HR of 4.7 (95% CI, 1.2–7.0). The median OS of the patients with TLR < 1 was not reached after 114 months, while 1–2.3 and >2.3 had median OS of 55 (95% CI, 27.2–82.9) and 13 months (95% CI, 6.1–19.9), respectively [110]. 

The FDG-PET-derived tumor burden, measured as MTV and total lesion glycolysis (TLG), was also evaluated in NET patients. In a study of 190 NET patients, patients with an MTV higher than 4.83 mL had a lower median OS compared to those with a lower MTV (29.7 months vs. not reached, HR 4.1, 95% CI 2.25–7.49, *p* < 0.0001). MTV and TLG prognosticated OS and PFS in a multivariate analysis (*p* < 0.001), whereas the SUVmax of the index lesion was not a statistically significant predictor. In the FDG-positive group, patients with MTV > 33 mL had worse OS compared to those with MTV <33 mL (28.0 months vs. 54.2 months, HR 1.94, 95% CI 1.11–3.38, *p* = 0.01) [77]. 

**Figure 1:**
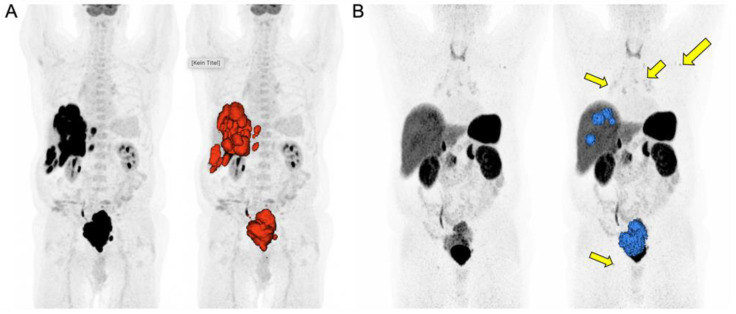
SSTR and FDG-PET of a patient with neuroendocrine tumor. A 71-year-old male patient diagnosed with a grade 3 neuroendocrine tumor derived from the rectum with a Ki-67 index of 80% and histopathologically proven high SSTR-2A expression. The patient was referred to nuclear medicine for PRRT after carboplatin, etoposide, and 5FU therapies. On the MIP images, all lesions showed intense uptake on FDG-PET (**A**), while mild to moderate SSTR expression was detected on SSTR-PET (**B**). Additionally, mild uptake secondary to inflammatory processes in bilateral hilar-mediastinal left axillary and right inguinal lymph nodes were observed ((**B**), arrows). Therefore, the patient was not referred to PRRT.

Given the number of studies that demonstrated the value of SSTR-PET and FDG-PET features as promising imaging biomarkers, Chan et al. recommended the simultaneous assessment of FDG- and SSTR-PET and proposed a five-point NETPET scoring system for the visual assessment of metastatic NETs. In this study, P2-4 was defined as the predominant SSTR-positive/FDG-positive disease, P1 represented SSTR-positive/FDG-negative disease, and P5 represented significant SSTR-negative/FDG-positive disease. NETPET grade was found to be significantly associated with WHO 2010 histological grade (Spearman’s correlation, r = 0.57, *p* < 0.00001) [111]. Moreover, NETPET grade was found to be a significant predictor of overall survival in both univariate and multivariate analyses of GEP-NET and bronchial NETs (*p* < 0.001 and *p* < 0.05, respectively) [112]. The prognostic importance of the NETPET score was recently analyzed in a multi-center study of 319 GEP-NET patients. The median overall survival (time to progression) was 101.8 (25.5) months for P1, 46.5 (16.7) months for P2–4, and 11.5 (6.6) months for P5 [112]. Figure 1 shows an image example of a patient (NET derived from the rectum) with FDG-negative disease with a good therapy response to PPRT.

A dual-tracer approach with FDG and SSTR-PET was also found to be helpful in preoperative staging, prognostication, and risk stratification of pulmonary carcinoid tumors and may affect surgical management. FDG SUVmax and TLR were significantly higher in atypical carcinoids, whereas SSTR SUVmax, TLR, and the ratio of SUVmax in SSTR-PET to FDG-PET were significantly higher in typical carcinoids. Lococo et al. reported that a ratio of ≥1.19 between the SUVmax on SSTR-PET and the SUVmax on FDG-PET can differentiate typical from atypical carcinoid with a sensitivity and specificity of 82.6% and 90%, respectively (AUC, 0.90; 95% CI, 0.79–1.00, *p* = <0.001) [113].

### 3.7. SSTR-PET-Derived Tumor Volume in the Context of PRRT

**Figure 2:**
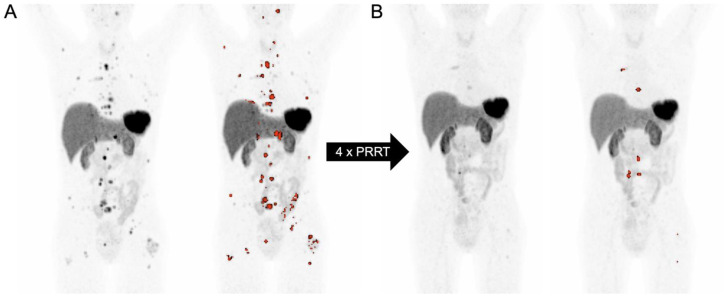
Response in SSTR-derived tumor volume of a patient undergoing PRRT. A 46-year-old female patient was diagnosed with a grade 2 neuroendocrine tumor derived from the rectum with a Ki-67 index of 10%. After surgery, octreotide, capecitabine + oxaliplatine, PRRT with the cumulative activity of 5.6 GBq of 90Y-DOTATATE and 14.8 GBq of 177Lu-DOTATOC, SIRT, and everolimus therapy, the patient received 4 cycles of additional PRRT with 177Lu-DOTATATOC. On the baseline MIP images, one liver metastases and multiple bone metastases with intense SSTR expression were seen (**A**). After 4 cycles of PRRT, the liver metastasis was no longer detected, and the number of SSTR-expressing bone metastases was declining (**B**).

The prognostic impact of SRE-TV in NET patients has also been confirmed in the context of patients undergoing PRRT, where low SRE-TV and high SUVmean in the lowest uptake lesions were associated with longer OS [82]. Durmo et al. were able to show an association between high SRE-TV and the likelihood of response to PRRT as well as OS (HR 12.76, *p* = 0.01); Pauwels et al. additionally showed a prognostic impact of the inflammation-based index, a composite metric using CRP and albumin levels [88,104]. Interestingly, in this cohort, changes in volumetric parameters derived from SSTR-PET from baseline to interim imaging were not associated with patient outcome [88]. Ebbers et al. showed that both baseline (*p* = 0.03) and post-PRRT SRE-TV (*p* = 0.01) are predictive of disease progression, parametrized by time to new treatment [80]. Figure 2 shows an image example of a patient with a neuroendocrine tumor who underwent PRRT.

## 4. Discussion

The past years have seen a steep increase in publications assessing the prognostic implications of biomarkers derived from SSTR-PET, with a focus on uptake intensity and SRE-TV. Unsurprisingly, the high amount of data extractable from SSTR-PETs lends itself to accurate risk stratification of NET patients undergoing both variable as well as SSTR-directed treatments, such as PRRT and SSA. Furthermore, high SRE-TV is associated with hormonal excess, both clinically and biochemically [86,90,97].

Due to the straightforward quantification possibility, the SUVmax has been employed to monitor the disease or assess PRRT eligibility. However, the SUVmax originates from single voxel measurement and can, therefore, be prone to errors. In addition, it neglects the intra- and inter-lesional heterogeneity of tumor manifestations. Especially in NETs, a discordance in SSTR expression between the primary tumor and the metastases has been described [114]. Therefore, the analysis of all tumor manifestations and the integration of measurements in a tumor volume should overcome this limitation. Corroborating this idea, SSTR-PET-derived tumor volume has been identified as a prognosticator of both PFS and OS [115].

Distinct metastases may harbor different cellular clones with varying SSTR expression levels. Miller et al. reported that 35.3% of GEP-NET cases show a Ki-67 discordance between primary tumors and metastases [116]. Also, the tumor cells of an individual patient can have different characteristics regarding their genome, epigenome, and transcriptome. These differences may result in variations in metabolism, proliferation, and metastatic potential, leading to clonal selection when treated with drugs that target only one particular subtype. As a result, temporal, intra-, and inter-tumoral heterogeneity can occur. This may also explain the high rate of progressive disease observed in 20–30% of NET patients treated with PRRT, despite the fact that tumor lesions received up to 250 Gy [117], which usually leads to treatment response. The SSTR-negative disease may indicate a metabolically active disease with increased FDG uptake, which indicates a poor prognosis. To assess this well-known flip-flop phenomenon (e.g., a lesion that is positive on FDG-PET and negative on SSTR-PET), dual imaging with SSTR- and FDG-PET can be performed. Dual imaging may help to assess disease heterogeneity at the whole-body level, but is typically only performed in selected patients with advanced NETs. Use of FDG and SSTR-PET may be recommended in cases of high Ki-67, lesions with low or no SSTR tracer uptake, and clinical or radiological evidence of disease progression despite low Ki-67 [106]. 

Apart from its prognostic importance, FDG-PET may help to select the patients diagnosed with G3 NETs, who will benefit from the combination therapy of PRRT with radiosensitizing fluorouracil (5FU) [118]. Patients with FDG-avid tumors, if they have high SSTR expression, may benefit from combination therapies, so exclusion from PRRT is not necessary [118]. Dual PET evaluation of pancreatic NETs can potentially be helpful in the selection of SSA/PRRT or chemotherapy. Irrespective of SSTR expression, patients with high FDG uptake on any side of the disease may be offered chemotherapy as first-line treatment, but may also be considered for external beam radiotherapy of isolated bone or soft tissue lesions that could be treated with low morbidity [72]. The clinical relevance of preoperatively distinguishing between G1 and G2 pancreatic NETs could be also of major value in cases where there is no consensus on the surgical indication, that is, small tumors (<2 cm). In such cases, the complementary information provided by 18F-FDG-PET could help determine the indication for surgery and the extent of surgery [47].

Morphological response assessment has several limitations for NET patients. Changes in size may not be an optimal surrogate marker for a response since the size of many lesions increases or remains unchanged as tumors become fibroid, cystic, or mixed. Secondly, contrast enhancement of lesions can also be inconclusive due to technical differences (e.g., the timing of iv contrast administration) or changing physiology [119]. In such instances, patients may be miscategorized as having a poor response or progressive disease, resulting in the inappropriate cessation of effective therapy. Moreover, biochemical response evaluation may not be efficient in non-functional tumors or may be inconclusive in the case of mixed responses. 

In contrast to morphological imaging modalities, SSTR-PET imaging is a useful tool to evaluate tumor responses with molecular imaging. However, several limitations must be considered when using SSTR-PET for patient monitoring. For example, it was found that therapy with long-acting SSA causes approximately a 25% and 20% reduction in 68Ga-DOTATATE uptake in the spleen and liver, respectively, whereas increased uptake within metastases was seen in 90% of the cases. These findings may lead to false assessments of disease response [120]. Furthermore, the decision to use SSTR-PET was based on the initial histopathological characteristics of the tumor. However, SSTR-PET may not effectively detect disease progression in patients with suspected higher-grade tumors due to temporal heterogeneity, especially when clinical findings of progression are present. Therefore, functional, and metabolic evaluation of the disease during therapy or follow-up at the whole-body level may also play a decisive role in the determination of therapy management. Patients with FDG-negative NET initially may become FDG-positive during follow-up. Nilica and colleagues categorized 66 NET patients into four different groups according to baseline and follow-up FDG-PET findings in addition to SSTR-PET during follow-up after PRRT as follows: FDG-negative patients both initially and during follow-up, FDG-positive patients both initially and during follow-up, initially FDG-negative patients who turned FDG-positive during follow-up, and initially FDG-positive patients who turned FDG-negative during follow-up. The first category was associated with a better response rate, and FDG-positive tumors correlate strongly with a higher risk of progression [44]. 

The prostate-specific membrane antigen (PSMA) is a marker for prostate cancer cells, which enables the PET-based imaging of the tumor burden in patients with prostate cancer. PSMA-PET has shown superior diagnostic performance both in the initial stages of high-risk prostate cancer and in biochemically recurrent prostate cancer [121,122]. Following a theranostic concept, the PSMA molecule can also be targeted for PET imaging and radionuclide therapy [123]. It is worthwhile to analyze the current concepts of tumor volume and response assessment in prostate cancer, as similar concepts could be used for NET patients and SSTR-PET. For PSMA-PET imaging, a comprehensive framework (PROMISE 2.0) defining the measurable features has been proposed, which provides a foundation to enable standardized reporting of the tumor extent analogous to the TNM format [124]. In addition, frameworks built on top of this foundation have been proposed, which are tailored for limited disease (PPP) and extensive disease (RECIP) [125,126]. The RECIP framework integrates the appearance of new lesions and total tumor volume to make an integrated judgment on the response of the patient. A similar approach would be valuable for patients with extensive disease manifestations of neuroendocrine tumors, as radiological frameworks like RECIST or FDG-PET frameworks like PERCIST are often not applicable to this cohort of patients [127,128]. However, it must be acknowledged that unspecific fluctuations in uptake due to, e.g., SSA medication may pose challenges to these assessments. Additionally, the non-linear correlation between SSTR expression and SUV leads to an underestimation of SSTR expression at the high end, further hampering uptake-based response assessments. This may be addressed by the use of alternative markers, such as the net influx rate or tumor–blood ratio [129,130].

Current studies investigating SSTR-PET-derived biomarkers face some limitations. It is worth pointing out that all volumetric studies were of retrospective nature and did not overtly influence treatment decisions in prospective studies. A couple of clinical and methodological questions still remain unanswered. Different methodologies across studies limit the comparability of study results, and the published data do not yet allow for a final conclusion as to which definition for lesion segmentation is superior, even if there is some evidence that a 30% threshold of the local SUVmax is highly correlated with morphological tumor volume. In addition, it has yet to be assessed in a prospective fashion whether patients with a high SRE-TV and consecutively high distribution volume of SSTR-directed agents may benefit from treatment escalation in terms of dose or activity. Another challenge for prognosticating the outcome of patients with neuroendocrine tumors is the availability of reliable endpoints. Due to the relatively long life expectancy, the use of overall survival as an endpoint is limited, as the events are relatively rare, and the association of death with disease progression is often not given. Other endpoints like PFS are difficult to obtain in a retrospective setting, which limits the reliability and transferability of the results.

Future trends of SSTR-PET-derived biomarkers comprise the standardization of volumetric measurements and the integration of response assessment frameworks, like those that have been proposed for other tracers and cancer entities, for example, the RECIP framework for PSMA-PET. In addition, given the high costs of PRRT, it is likely that comprehensive nomograms or response prediction frameworks will be developed. Such approaches would enable physicians to make treatment decisions on a sounder foundation by selecting only patients for PRRT who are likely to benefit from the procedure.

## 5. Conclusions

SSTR-PET-derived quantitative metrics like tumor volume and uptake are promising biomarkers that might improve the management of patients with neuroendocrine tumors. However, the lack of standardization currently limits their utilization in the clinical routine. Prospective trials investigating SSTR-PET-derived biomarkers, especially in the context of PRRT, are warranted.

## Abbreviations

SSTR	Somatostatin receptor
NET	Neuroendocrine tumor
SRS	Somatostatin receptor scintigraphy
SSTR-PET	Somatostatin receptor positron-emission tomography
PET	Positron-emission tomography
SUV	Standardized uptake value
MRI	Magnetic resonance imaging
CT	Computed tomography
PRRT	Peptide radionuclide receptor therapy
PSMA	Prostate-specific membrane antigen
SRE-TV	Somatostatin receptor-expressing tumor volume
PFS	Progression-free survival
OS	Overall survival
SSA	Somatostatin analogs
FDG	Fluorodeoxyglucose
MTV	Metabolic tumor volume
PERCIST	PET response criteria in solid tumors
SD	Standard deviation
SUVmax	Maximum standardized uptake value
SUVmean	Mean standardized uptake value
pNET	Pancreatic neuroendocrine tumor
TLR	Tumor-to-liver ratio
IBI	Inflammation-based index
G1/2/3	Grade 1/2/3
CA9	Cancer antigen 9
GLUT1	Glucose transporter 1
Gy	Gray
5-FU	5-fluorouracil
PROMISE	Prostate cancer molecular imaging standardized evaluation
TNM	Tumor node metastasis
PPP	PSMA PET progression criteria
RECIP	Response evaluation using PSMA PET/CT in patients with metastatic castration-resistant prostate cancer
